# Exploring Nurses’ Perspectives on the Use of Artificial Intelligence Chatbots for Mental Health Support: A Cross-Sectional Study in Greece

**DOI:** 10.3390/nursrep16040133

**Published:** 2026-04-13

**Authors:** Paschalina Lialiou, Aglaia Katsiroumpa, Parisis Gallos, Olympia Konstantakopoulou, Ioannis Moisoglou, Olga Galani, Maria Tsiachri, Petros Galanis

**Affiliations:** 1 Department of Digital Systems, University of Piraeus, 18534 Piraeus, Greece; 2 Faculty of Nursing, National and Kapodistrian University of Athens, 10679 Athens, Greece; olykonstant@nurs.uoa.gr; 3 Clinical Epidemiology Laboratory, Faculty of Nursing, National and Kapodistrian University of Athens, 11527 Athens, Greece; aglaiakat@nurs.uoa.gr (A.K.); mtsiachri@nurs.uoa.gr (M.T.); pegalan@nurs.uoa.gr (P.G.); 4 Faculty of Nursing, University of West Attica, 12243 Egaleo, Greece; parisgallos@uniwa.gr (P.G.); ogalani@uniwa.gr (O.G.); 5 Faculty of Nursing, University of Thessaly, 41500 Larissa, Greece; iomoysoglou@uth.gr

**Keywords:** artificial intelligence, nurses, nursing, mental health, conversational agents, chatbots, acceptance, fear, attitudes

## Abstract

**Background/Objectives**: Artificial intelligence (AI) has transformed healthcare delivery by revolutionizing the offering opportunities in prognosis, diagnosis, personalized treatment, and improving patient outcomes. However, little is known about the nurses’ attitudes toward the integration of AI-driven conversational technology and AI chatbots into clinical practice. The aim of our study was to investigate nurses’ attitudes regarding the use of AI chatbots as a tool for mental health support. Additionally, the study aimed to evaluate their levels of acceptance and fear toward AI, while examining the influence of demographic variables on these attitudes. **Methods**: A cross-sectional study was conducted. We employed the Artificial Intelligence in Mental Health Scale (AIMHS) to measure attitudes toward the use of AI-powered chatbots for mental health support. Additionally, we utilized the Attitudes Towards Artificial Intelligence Scale (ATAI) to assess nurses’ levels of acceptance and fear regarding artificial intelligence. **Results**: Technical advantages score in the AIMHS reflected low positive attitudes toward the technical aspect of AI chatbots for mental health support, while personal advantages score showed moderate positive attitudes toward the personal aspect of chatbots. ATAI scores indicated a moderate level of acceptance and fear toward AI. Results from multivariable analysis showed that increased age (b = 0.011, *p*-value = 0.018) and increased daily engagement with social media and websites (b = 0.058, *p*-value = 0.002) were significantly associated with more favorable technical attitudes towards AI-based mental health chatbots. Also, male nurses exhibited significantly more favorable attitudes toward AI-based mental health chatbots in terms of perceived personal benefits (b = 0.548, *p*-value < 0.001). Higher levels of digital technology competence were significantly associated with greater acceptance of artificial intelligence (b = 0.164, *p* = 0.032). Additionally, male nurses reported significantly higher acceptance of AI compared to their female counterparts (b = 1.587, *p* < 0.001). We found that lower financial status was significantly associated with heightened fear of AI (b = −0.329, *p* < 0.001). **Conclusions**: Nurses generally held moderately positive attitudes toward both AI-based mental health chatbots and AI more broadly. Several demographic factors were found to significantly influence these attitudes.

## 1. Introduction

In 2019, the WHO claimed that 970 million people globally struggle with mental disorders, with anxiety and depression being the most common [1]. Due to the complex content of mental disorders, many aspects of life are affected, including relationships, family, friends, community, school performance and work life. Moreover, people who suffer from a mental disorder face a high risk of suicide and their productivity is reduced. In this context, the impact of mental disorders on the health care system and economy is tremendous. It is estimated that approximately 70% of individuals with mental illness do not receive formal treatment [2], and many others do not seek care due to a low perceived need and attitudinal barriers such as stigma or financial constraints [3]. Stigma is closely linked to mental illness and constitutes a significant barrier for seeking help, leading to negative attitudes and intentions toward mental health services among individuals [3,4].

Conversely, the global deficit of mental healthcare providers presents a significant challenge to service delivery [5]. A worldwide disparity in the distribution of health care providers creates substantial barriers to the equitable delivery of mental healthcare. Findings also support that there is a shortage of mental health nurses [6,7]. Nursing workforce shortages have long been addressed with exacerbating attrition and projected workforce shortfalls [8]. Because nursing is inherently relational and involves significant daily workplace stressors, practitioners require advanced cognitive and emotional competencies to maintain effective self-regulation [9]. Nurses frequently encounter challenges inherent to providing interpersonal care for patients in acute emotional distress, including those exhibiting self-harm or suicidal behaviors, while simultaneously managing clinical aggression and interpersonal conflicts [10]. As a consequence, their own mental integrity is impacted, resulting in lower mental well-being than the general population norms [10].

All the aforementioned issues related to the increased need for mental health support among the population, such as stigma, the shortage of mental health nurses, and the challenges to access in mental health services, were exacerbated during the COVID-19 pandemic. The COVID-19 pandemic exposed significant deficiencies in mental health accessibility, as traditional service delivery was severely constrained. Consequently, rapid interventions become necessary to bypass these barriers and enhance engagement with psychosocial treatment in both adult and youth populations [11]. Driven by the rapid expansion of the data ecosystem, significant advances in artificial intelligence and machine learning have emerged. This landscape has gained substantial attention for its potential to enhance diagnostic accuracy, streamline clinical workflows, and address healthcare disparities [12]. Large language models (LLMs) are defined as pretrained models (PLMs) characterized by vast parameter counts and training on massive amounts of datasets. Due to their capacity for solving complex tasks, they have become a focal point of AI research and serve as the foundation of generative AI applications. By understanding and generating natural language, images, and videos, LLMs facilitate sophisticated human–machine interactions with transformative potential for medical diagnostics [12]. Furthermore, LLMs are being rapidly integrated into disease classification, medical inquiry, demonstrating high accuracy across radiology, psychiatry and neurology [13,14].

In the era of LLMs, conversational AI is expanding rapidly. These AI-driven technologies have the potential to provide instant, interactive and context-aware access to clinical recommendations [15]. Often referred to as conversational or relational agents, these chatbots engage in dialogue with human users, thereby enhancing the engagement of healthcare professionals providing real-time clinical guidance [16,17]. The digital mental health industry bridges existing service gaps by incorporating artificial intelligence to create AI-guided products. By performing human-like physical and cognitive tasks, these technologies enable automation and AI-supported decision making, which is typically reviewed by domain experts before implementation in a patient treatment plan [18]. Furthermore, AI capabilities support both clinicians and patients, improving population health outcomes and transforming mental healthcare by increasing patient retention, optimizing work–life balance and reducing costs [17,19].

Previous studies have explored the potential of AI-powered chatbots in mental health [11,16,20,21]. For nurses, intelligent conversational agents offer opportunities to augment nursing practice and care planning, support patient education, empower individuals, and manage health outcomes [22,23]. While AI chatbots show promise in diagnostic accuracy and clinical decision-making, human clinicians currently excel in holistic clinical abilities—skills that require experience, contextual knowledge, and the capacity to provide nuanced responses [22]. Prior research reveals that conversational agent-based interventions are feasible, acceptable, and effective regarding physical function, healthy lifestyle, mental health and psychosocial outcomes [24,25]. Conversely, others suggest that Retrieval Augmented Generation (RAG)-enabled AI conversational systems may generate misleading information due to bias, outdated training data, and misinformation embedded in public resources [15]. Consequently, there is a need for ongoing research to examine whether AI technology can adequately substitute for nursing expertise while prioritizing patient safety, privacy, and equitable access to care.

As LLMs and AI-driven chatbots are increasingly explored as problem-solving aids across healthcare domains, access to medical information is expanding rapidly. The scientific community is working systematically to enhance the capacity of these models while addressing critical issues such as ethical concerns, privacy risks, model hallucination, and regulatory fragmentation. As technology evolves, confidence in medical technology among healthcare professionals and patients is being shaped by AI’s ability to remain evidence-based, unbiased and equitable [19]. More precisely, the widespread adoption of AI chatbots in clinical settings remains a subject of debate [19,22].

Recently, several surveys explored healthcare professionals’ attitudes toward artificial intelligence in general. But just a few studies have examined nurses’ perspectives, beliefs, and attitudes toward mental health chatbots. Furthermore, few studies have explored the specific factors that shape these perspectives, which ultimately influence the adoption or rejection of this digital technology. Also, limited studies have examined attitudes towards AI-enabled mental health chatbots using validated measurement instruments. As frontline healthcare providers, nurses play a key role in shaping healthcare policies and the overall function of the healthcare system; therefore, this study aims to address this research gap and to highlight the critical role of nurses’ attitudes in the adoption of mental health chatbots in healthcare. More precisely, based on the aforementioned status in the international literature, this study aims to contribute to the literature by

(i) Applying the Artificial Intelligence in Mental Health Scale (AIMHS) and the Attitudes Toward Artificial Intelligence Scale (ATAI) in a nationwide sample of nurses;
(ii) Revealing nurses’ attitudes regarding the use of AI-driven chatbots for mental health support;
(iii) Investigating how demographic and digital competence characteristics of nurses affect their attitudes towards the above technologies.

We used two scales, i.e., the AIMHS and the ATAI, since we want to explore both nurses’ attitudes toward mental health chatbots and attitudes toward AI in general. Since the ATAI assesses individuals’ acceptance and fear of AI, we considered that we should use a specific tool to measure nurses’ attitudes toward mental health chatbots. In this context, we established the following research hypotheses:

(i) Demographic characteristics such as gender, age, and financial status are associated with nurses’ attitudes toward mental health chatbots.
(ii) Daily time spent on social media and/or websites and proficiency in digital technologies are associated with nurses’ attitudes toward mental health chatbots.
(iii) Demographic characteristics such as gender, age, and financial status are associated with nurses’ acceptance and fear of AI.
(iv) Daily time spent on social media and/or websites and proficiency in digital technologies are associated with nurses’ acceptance and fear of AI.

To examine our research hypotheses, we used the AIMHS to measure nurses’ attitudes toward mental health chatbots, and the ATAI to measure nurses’ acceptance and fear of AI.

## 2. Materials and Methods

### 2.1. Study Design

A cross-sectional study was conducted in Greece, with data collection taking place in October 2025 via an online survey. This survey used an independent sample that has not been used in any previous publications by the author group. A new study protocol was developed specifically for this research and ethical approval was obtained from the Ethics Committee of the Faculty of Nursing at the National and Kapodistrian University of Athens. The questionnaire was distributed using Google Forms through nursing-focused groups on social media platforms such as Facebook and LinkedIn, resulting in a convenience sample. Participants were recruited exclusively for this study and the data collected are original (not exported from previous survey cohorts). Eligibility criteria for participation included: (a) being a registered nurse, (b) having an active account on at least one social media platform, (c) engaging with social media and/or the internet for a minimum of 30 min daily to ensure sufficient exposure to digital technologies, and (d) providing informed consent prior to completing the survey. The study adhered to the Strengthening the Reporting of Observational Studies in Epidemiology (STROBE) guidelines [26].

The sample size was calculated using G*Power software (version 3.1.9.2). The following parameters were applied in the computation: (a) a Type I error probability of 5%, (b) statistical power of 95%, (c) five independent variables, and (d) a small effect size (f^2^ = 0.05) for the association between each independent and dependent variable. Based on these criteria, the required sample size was determined to be 262 nurses.

### 2.2. Measurements

The demographic variables examined were: (a) gender (male or female), (b) age, treated as a continuous variable, (c) self-assessed financial status on a scale from 0 (very poor) to 10 (very good), (d) daily time spent on social media and/or websites (continuous variable), and (e) self-reported proficiency in digital technologies—including smartphones, personal computers, AI applications, and tablets—measured on a scale from 0 (very poor) to 10 (very good).

Nurses’ attitudes toward mental health chatbots powered by artificial intelligence were evaluated using the Artificial Intelligence in Mental Health Scale (AIMHS) [27]. This instrument comprises five items distributed across two dimensions: technical advantages (2 items) and personal advantages (3 items). Participants responded using a 5-point Likert scale ranging from 1 (strongly disagree) to 5 (strongly agree). Factor scores were computed by averaging the item responses within each dimension, yielding scores between 1 and 5, with higher values reflecting more favorable attitudes toward AI-based mental health chatbots. Two negatively worded items were reverse-coded prior to analysis. The validated Greek version of the AIMHS [27] was employed in this study. Internal consistency was deemed very good, with Cronbach’s alpha coefficients of 0.788 for the technical advantages subscale and 0.896 for the personal advantages subscale.

To assess nurses’ acceptance and fear of artificial intelligence, we employed the Attitudes Towards Artificial Intelligence Scale (ATAI) [28]. This instrument comprises five items: two items evaluate acceptance of AI, while three items assess fear related to AI. Responses are recorded on an 11-point Likert scale ranging from 0 (strongly disagree) to 10 (strongly agree). The overall score is calculated as the mean of all item responses, yielding a range from 0 to 10. Higher scores on the acceptance subscale reflect greater acceptance and more favorable attitudes toward AI, whereas higher scores on the fear subscale indicate increased fear and more negative attitudes. For this study, we utilized the validated Greek version of the ATAI [29]. Internal consistency was very good, with Cronbach’s alpha coefficients of 0.857 for the acceptance subscale and 0.715 for the fear subscale.

### 2.3. Ethical Issues

Participation was entirely voluntary and anonymous. Prior to enrollment, all potential participants received a comprehensive participant information about the study’s aims and procedures, and an informed consent form. All participants gave their written consent before taking part. The study was conducted online using a web-based survey platform. The participant information sheet, which detailed the study’s purpose, procedures, potential risks, and data protection principles, was provided on the first page of the survey. Immediately following this information, participants were presented with an electronic informed consent form. They were required to read the information and indicate agreement by selecting the ‘I consent to participate’ option before gaining access to the survey questions. No data were recorded unless participants provided this electronic written consent. The research was conducted in compliance with all ethical principles, following the Declaration of Helsinki [30]. Ethical approval for the study was granted by the Ethics Committee of the Faculty of Nursing at the National and Kapodistrian University of Athens (Protocol No. 01, dated 14 September 2025).

### 2.4. Statistical Analysis

Categorical variables were described using frequencies and percentages, whereas continuous variables were summarized through means, standard deviations (SDs), medians, and interquartile ranges. The normality of continuous variable distributions was evaluated using the Kolmogorov–Smirnov test and Q–Q plots, both of which supported the assumption of normality. Demographic characteristics were treated as independent variables, while attitudes toward AI-based mental health chatbots, AI acceptance, and AI-related fear were considered as dependent variables. Given the continuous and normally distributed nature of the dependent variables, both simple and multivariable linear regression analyses were conducted. Initially, bivariate linear regressions were performed, followed by the development of final multivariable models. Multicollinearity was assessed using variance inflation factors (VIFs), with values exceeding 5 indicating potential multicollinearity concerns. Model assumptions were verified by inspecting residual histograms for normality, and residual-versus-predicted scatterplots for homoscedasticity and linearity [31]. The findings are presented as unadjusted and adjusted beta coefficients, along with 95% confidence intervals (CIs) and *p*-values. Furthermore, Pearson’s correlation coefficient was employed to examine the association between AIMHS and ATAI scores. *p*-values less than 0.05 were considered statistically significant. We used the IBM SPSS 28.0 (IBM Corp., released 2021. IBM SPSS Statistics for Windows, Version 28.0. Armonk, NY, USA: IBM Corp) for the statistical analysis.

## 3. Results

### 3.1. Demographic Characteristics

The final sample comprised 276 nurses, with the majority being female (81.5%). The mean age of participants was 42.94 years. The average self-perceived financial status was 5.97. Participants reported a mean daily duration of social media and/or website usage of 3.07 h. The mean score for self-assessed digital competence was 7.63. A detailed overview of the demographic characteristics is provided in Table 1.

### 3.2. Study Scales

The mean score on the technical advantages factor was 1.76, while on the personal advantages factor was 3.26. The mean acceptance score was 5.15, while the mean fear score was 5.72. Detailed descriptive statistics for the study scales are presented in Table 2.

We found a statistically significant positive correlation between the technical advantages factor and acceptance of AI (r = 0.533, *p*-value < 0.001). Moreover, we found a statistically significant negative correlation between the technical advantages factor and fear of AI (r = −0.148, *p*-value = 0.014). A positive correlation between the personal advantages factor and acceptance of AI was identified (r = 0.537, *p*-value < 0.001). Detailed correlation results are presented in Table 3.

### 3.3. Dependent Variable: Artificial Intelligence in Mental Health Scale

Table 4 displays the findings from the linear regression analysis, wherein the “technical advantages” dimension of the AIMHS served as the dependent variable. In the final multivariable model, both older age (b = 0.011, 95% CI: 0.002–0.020, *p*-value = 0.018) and increased daily engagement with social media or websites (b = 0.058, 95% CI: 0.021–0.095, *p*-value = 0.002) were significantly associated with more favorable attitudes towards AI-based mental health chatbots. In practical terms, a one-year increase in age corresponded to an estimated 0.011-unit increase in the score on the technical advantages factor. Moreover, a one-hour increase in daily engagement with social media or websites corresponded to an estimated 0.058-unit increase in the score on the technical advantages factor. Diagnostic checks confirmed that the model met the necessary assumptions: residuals were normally distributed (see Supplementary Figure S1), homoscedasticity and linearity were evident (see Supplementary Figure S2), and variance inflation factors (VIFs) revealed no multicollinearity among the independent variables.

Table 5 presents the results of the linear regression analysis, with the personal advantages dimension of the AIMHS serving as the dependent variable. In the final multivariable model, male nurses exhibited significantly more favorable attitudes toward AI-based mental health chatbots in terms of perceived personal benefits (b = 0.548, 95% CI: 0.277–0.820, *p*-value < 0.001). In practical terms, male nurses scored, on average, 0.548 units higher on the personal advantages factor compared to female nurses. Diagnostic assessments confirmed that the assumptions underlying linear regression were satisfied: residuals were normally distributed (see Supplementary Figure S3), homoscedasticity and linearity were observed (see Supplementary Figure S4), and VIFs suggested the absence of multicollinearity among the independent variables.

### 3.4. Dependent Variable: Attitudes Toward Artificial Intelligence Scale

Table 6 displays the results of the linear regression analysis, with the acceptance dimension of the ATAI serving as the dependent variable. In the final multivariable model, higher levels of digital technology competence were significantly associated with greater acceptance of artificial intelligence (b = 0.164, 95% CI: 0.014–0.313, *p* = 0.032). In practical terms, a 1-unit increase in competence in digital technologies corresponded to an estimated 0.164-unit increase in the score on the acceptance factor. Additionally, male nurses reported significantly higher acceptance of AI compared to their female counterparts (b = 1.587, 95% CI: 0.993–2.181, *p* < 0.001). In practical terms, male nurses scored, on average, 1.587 units higher on the acceptance factor of the ATAI compared to female nurses. Model diagnostics indicated that the assumptions of linear regression were adequately met: residuals were normally distributed (see Supplementary Figure S5), homoscedasticity and linearity were observed (see Supplementary Figure S6), and VIFs confirmed the absence of multicollinearity among the independent variables.

Table 7 outlines the findings of the linear regression analysis, with the fear dimension of the ATAI utilized as the dependent variable. In the final multivariable model, lower financial status was significantly associated with heightened fear of artificial intelligence (b = −0.329, 95% CI: −0.467 to −0.190, *p* < 0.001). In practical terms, a 1-unit increase in financial status corresponded to an estimated 0.329-unit decrease in the score on the fear factor. Furthermore, male nurses reported significantly greater levels of fear toward AI compared to their female counterparts (b = 0.772, 95% CI: 0.231–1.312, *p* = 0.005). In practical terms, male nurses scored, on average, 0.772 units higher on the fear factor of the ATAI compared to female nurses. Diagnostic evaluations confirmed that the assumptions of linear regression were satisfactorily met: residuals exhibited a normal distribution (see Supplementary Figure S7), homoscedasticity and linearity were observed (see Supplementary Figure S8), and VIFs demonstrated no evidence of multicollinearity among the independent variables.

## 4. Discussion

This study aimed to identify nurses’ attitudes regarding the use of AI-driven mental health chatbots. Findings highlight the levels of acceptance and the fear toward AI integration in clinical practice, while offering several insights into the relationships among nurses’ familiarity with AI, their beliefs, their affective attitudes and their behavioral intention regarding mental health support.

Overall, nurses exhibited positive attitudes toward the utilization of AI-driven chatbots. Generally, nurses did not perceive substantial technical advantages of AI technology; however, they reported more personal benefits from the use of AI mental health chatbots. As illustrated by the results, nurses who recognize more technical advantages of artificial intelligence feel more comfortable and less fearful of accepting AI applications. Conversely, nurses who are aware of numerous personal benefits of using AI chatbots also tend to experience higher levels of fear. We interpret these results as reflecting personal gains that AI provides to users, while concerns and controversy regarding AI still remain. We suggest that, while participants acknowledge that the adoption of AI-driven chatbots may support their personal goals, like career advancement, and individual benefits, they do not view their technological contribution to the nursing profession positively. These findings align with previous research suggesting that experience with technology influences the adoption and acceptance of AI in healthcare [32,33]. Although nurses perceive many advantages to the use of artificial intelligence, they remain wary of its shortcomings, such as explainability and ethical issues within the nursing profession [34,35].

Another important outcome of our survey was that participants who perceive more technical advantages of AI tend to have a higher acceptance of the technology. In other words, as belief in technical benefits increases, acceptance increases concomitantly. These results are consistent with prior research, which emphasizes the role of beliefs in behavioral intention [36,37,38], leading to a common conclusion. Furthermore, the existing literature underscores AI’s expanding role in nursing as a complementary tool to available intervention strategies. AI mental health chatbots can reduce short-term anxiety and depression levels compared to the nurse hotline, but further research should be facilitated to ensure that intelligent nursing practices evolve in alignment with clinical realities to improve patients’ safety and quality of care, emphasizing the importance of evidence-based, cautious and content-sensitive application [39,40]. AI is a highly versatile tool with prospects in the nursing profession for support in nursing routines, like writing nursing documents, handling high-risk nursing operations and physical exertion, and carrying out many nursing activities [39]. Even though the nurses still appear optimistic about AI’s potential, ensuring the human touch remains essential for patients with complex needs [41].

Another key issue is that higher levels of digital technology competence were significantly associated with greater acceptance of artificial intelligence. Additionally, many variations between the genders were noted. Male nurses reported significantly higher acceptance of AI compared to their female counterparts, while they reported significantly greater levels of fear. This is consistent with the findings of the previous literature [36], in which female nurses reported lower levels of familiarity with AI, stronger beliefs about AI’s role in their job and higher levels of anxiety toward AI compared to their male counterparts. Regarding the predominance of males in the intention to adopt AI, a plausible interpretation is that attitudes toward AI are shaped by individuals’ perceptions of their role within the nursing profession, work-related usage experience, digital literacy, and social influence. Although these findings suggest that technology competence matters, they may be secondary to intention to adopt AI and fear. Moreover, cognitive beliefs often explain more variance in intention than affective evaluations alone. The interpretation of these results indicates that beliefs not only have a direct effect on intention but also indirectly influence intention through their effect on attitudes. This mediating role reinforces the sequence, wherein facilitating conditions act as antecedents to beliefs, beliefs act as antecedents to attitudes, and both beliefs and attitudes contribute to the formation of behavioral intention with regard to integrating AI into nursing practice [36]. Furthermore, the convergence of the present findings with prior research enhances the robustness of the AI acceptance related to gender differences, culture, and levels of anxiety [32,42,43,44,45,46]. This may lead to the conclusion that significant gender differences may arise from deeply rooted personal experiences and beliefs related to self-esteem. Therefore, interpretations that consider the unique characteristics of AI acceptance are necessary.

Our study underscores the transformative role of AI-driven conversational technology in health care, particularly in mental care. Within the specific context, mental health chatbots improve the management of patient care through realistic stimulations, adaptive technologies, data monitoring, collaborative treatments, planning and timely adjustment to interventions [47,48,49]. Still, much more research is required on how AI chatbots could align with patient engagement in health care and resource optimization in health care settings. AI chatbots are capable of advancing mental health care through enhanced accessibility and personalized interventions [50]. This approach allows the individualization of care, enhancing cultural sensitivity, optimizing outcomes, and contributing to responsive and effective mental health care [48]. However, careful consideration of all ethical implications and methodological concepts within the context of cultural adaptation is essential to reinforce the responsible deployment of AI-driven technology [51,52]. Such implications include ethical concerns about data privacy, security, and the need for strong safeguards, especially when handling sensitive mental health information [53,54]. On the other hand, trust deficits of AI and human hesitancy remain the two core barriers to widespread adoption, particularly in healthcare settings [55]. A conscientious and ethical integration of generative AI techniques is obligatory, ensuring a balanced approach that maximizes the benefits rather than the concerns [47,56]. Additionally, integration of AI-driven technology in competent use by all is obligatory. Educational interventions and innovative learning strategies enhance the development of a workforce that leverages the use of AI technology for efficient patient care [57]. More training programs within the academic settings, like web-based instruction, workshops and virtual reality clinical education, are recommended. Specifically in nursing education, the use of AI applications directly relates to informatics and technology competencies outcomes. Such outcomes are related to future reduction in nursing workload by performing certain tasks, thereby alleviating excessive burden and saving time [58]. Proactive leadership and policymakers are crucial to establish guidelines for the use, development and monitoring of AI systems in healthcare for ethical use of AI applications [57,58].

Overall, this study offers a novel perspective on the evolving literature regarding AI conversational agents in four significant ways. First, it expands the application of AI in mental health services (AIMHS) to a clinically active nursing population—a professional group on the frontline of healthcare delivery that remains underrepresented in empirical research. Second, by integrating both AIMHS and ATAI, the study provides a multidimensional assessment that captures both domain-specific perspectives, focused on mental health, and general AI attitudes within a unified framework. Third, the research situates attitudes within a clearly defined use case rather than examining AI in abstract terms, thereby enhancing the ecological validity and contextual relevance of the findings. Finally, the study moves beyond prior research by systematically analyzing the determinants of these attitudes. Specifically, it identifies cognitive, professional, and experiential factors that shape nurses’ attitudes, contributing to theoretical models of AI acceptance in high-stakes clinical environments.

## 5. Limitations

Overall, this study contributes to the body of evidence of existing research while highlighting the diverse perspectives and the need for ethical integration of AI chatbots in the nursing workforce. Nonetheless, this study has a bunch of limitations that should be considered when interpreting the abovementioned results. Firstly, this survey captures nurses’ attitudes at a single point in time. In addition, the data collected does not allow causal inferences regarding the determinants of attitudes towards AI-based mental health chatbots. Secondly, the study assessed self-reported attitudes rather than actual use of AI chatbots or genAI tools in clinical practice. Based on that, the reported attitudes may not fully reflect the real-world adoption behaviors and may lead to attitude–behavior gaps. Thirdly, participants were recruited through social media groups, resulting in a convenience sample that may overrepresent nurses who are more digitally engaged or technologically literate. Moreover, this survey captures participants’ attitudes and does not evaluate digital literacy or access to technological means. Overall, digital literacy and availability of digital services may reflect more skepticism, meaning that attitudes may be shaped by unfamiliarity, leading to further biasing results. Fourthly, although validated instruments were used to assess attitudes towards AI, the study did not directly measure participants’ prior exposure to specific AI systems such as LLM-based chatbots, which may influence their attitudes. Fifthly, since the study was conducted in a nursing sample, attitudes and beliefs may reflect concerns related to patient safety and ethics rather than efficiency. Finally, since the study was conducted in Greece, the findings may not be directly generalizable to other healthcare systems or cultural contexts. Future work includes the examination of nurses’ real-world interaction with AI-driven chatbots in clinical settings and exploring how AI literacy and targeted training influence the adoption of conversational AI tools in mental health care. Longitudinal studies could also provide insights into how attitudes toward AI evolve over time. Additionally, we should recognize that our cross-sectional study did not employ any experimental validation to examine our research hypotheses. Experimental studies in the future by comparing, for instance, nurses exposed to low or high levels of daily internet or social media use could add further evidence to this research field. Moreover, our sample was recruited through social media channels and professional networks, which may introduce a potential selection bias. Nurses who engage with these platforms tend to be more digitally active, professionally connected, or motivated to participate in research circulated in these online spaces. As a result, certain groups, such as nurses with limited internet access, lower social media use, or weaker ties to professional communities, may be underrepresented. This limitation should be considered when interpreting the findings and assessing their generalizability. We should mention that nurses were required to report a minimum of 30 min of daily internet or social media use to ensure a basic level of digital exposure. Nevertheless, given the lack of established standards defining what constitutes meaningful exposure, this criterion remains inherently arbitrary. Future research adopting alternative or multiple exposure thresholds may yield more nuanced insights into nurses’ attitudes toward mental health chatbots and artificial intelligence. A further limitation of our study is that the web-based distribution method does not allow for an accurate estimation of response bias. Because the survey link was disseminated online, it is not possible to determine how many nurses actually viewed the invitation but chose not to participate. Consequently, the true response rate cannot be calculated, and the representativeness of the sample relative to the broader nursing population remains uncertain. This limitation is inherent to open, web-based recruitment strategies and should be taken into account when interpreting the generalizability of our findings. Another limitation of this study concerns participants’ limited exposure to real-world chatbot use. Although the survey assessed nurses’ perceptions of mental health chatbots, most respondents had minimal or no direct interaction with such tools in clinical settings. As a result, their attitudes may reflect hypothetical beliefs or assumptions rather than informed evaluations based on practical experience. This gap between perceived and actual use is important, since hands-on engagement may substantially influence both acceptance and perceived usefulness of digital health technologies. Without real-world familiarity, nurses may overestimate risks, underestimate potential benefits, or rely on generalized views of AI shaped by media or professional discourse rather than concrete clinical encounters. Therefore, the findings should be interpreted with caution, and future studies incorporating experiential components, such as pilot implementation, guided demonstrations, or simulated chatbot interactions, may yield more valid insights into nurses’ attitudes toward mental health chatbots.

## 6. Conclusions

This study investigates nursing perspectives on AI-driven mental health chatbots and their broader attitudes towards AI technologies. While participants demonstrated favorable attitudes toward conversational tools, they reported significant concern regarding clinical implementation. Analysis indicates that demographic factors and digital competence statistically influence both AI acceptance and apprehension, confirming that technological familiarity constitutes a primary determinant of professional attitude. Consequently, enhancing AI literacy is critical; targeted training in generative AI is essential for navigating increasingly digitized healthcare environments.

On the other hand, the observed gender differences should be further interpreted with caution, given the imbalance in the nursing sample, and are therefore better considered as indicative of the need for further investigation. Additionally, the assessment of technological competence and affluence was based on self-reported measures, which are inherently subjective, and this limitation should be acknowledged while encouraging future studies to incorporate more objective indicators and include digitally excluded populations. Finally, further research is needed to explore nurses’ acceptance of AI across specific domains of mental care, in order to better understand the levels at which AI is perceived as acceptable or under what circumstances.

Also, this study leverages insights for policymakers and healthcare leaders to enable the integration of artificial intelligence into nursing practice, promoting patient safety and patient-centered care. There is an indisputable need to strengthen policy, education and resource allocation to support the sustainable integration of AI into clinical care. Notably, strict requirements must be considered for AI adoption, including patients’ perceptions, time efficiency, and preservation of clinician and patient autonomy. Many studies promote the design of relevant and sustainable education programs to support the adoption of AI within the mental health framework [48,59,60]. However, their effectiveness depends on many key issues, including ethical AI practices, security of patient data, and smooth integration into existing health care infrastructures [49]. Future initiatives should prioritize the responsible utilization of AI and the development of inclusive and accessible mental health chatbots supporting fair access to health care for all diverse populations.

## Figures and Tables

**Table 1:** Demographic characteristics of nurses (n = 276).

Characteristics	Mean	Standard Deviation	Median	Interquartile Range
Gender, n, %				
Females	225	81.5		
Males	51	18.5		
Age (years)	42.94	9.61	45	13
Financial status	5.97	1.56	6	2
Daily use of social media/websites (hours)	3.07	2.40	2	3.5
Competence in digital technologies	7.63	1.66	8	2

**Table 2:** Descriptive statistics for the study scales (n = 276).

Scale	Mean	Standard Deviation	Median	Interquartile Range
Artificial Intelligence in Mental Health Scale				
Technical advantages	1.76	0.69	2.00	0.69
Personal advantages	3.26	0.88	3.00	1.00
Attitudes Towards Artificial Intelligence Scale				
Acceptance	5.15	1.99	5.00	2.50
Fear	5.72	1.80	6.00	2.00

**Table 3:** Correlation analysis between the study scales (n = 276).

	Pearson’s Correlation Coefficient		
Scale	2	3	4
1. AIMHS (technical advantages)	0.522 **	0.533 **	−0.148 *
2. AIMHS (personal advantages)		0.537 **	0.001
3. ATAI (acceptance)			−0.221 **
4. ATAI (fear)			

* *p*-value < 0.05; ** *p*-value < 0.01. AIMHS: Artificial Intelligence in Mental Health Scale; ATAI: Attitudes Towards Artificial Intelligence Scale.

**Table 4:** Linear regression models with the score on the technical advantages factor of the Artificial Intelligence in Mental Health Scale as the dependent variable (n = 276).

Independent Variables	Univariate Models			Multivariable Model ^a^			VIF
	Unadjusted Coefficient Beta	95% CI for Beta	*p*-Value	Adjusted Coefficient Beta	95% CI for Beta	*p*-Value	
Males vs. females	0.096	−0.115 to 0.308	0.370	0.065	−0.149 to 0.279	0.551	1.068
Age	0.009	0.001 to 0.018	0.033	0.011	0.002 to 0.020	0.018	1.185
Financial status	0.031	−0.022 to 0.083	0.255	0.047	−0.008 to 0.102	0.090	1.126
Daily use of social media/websites (hours)	0.032	−0.002 to 0.066	0.069	0.058	0.021 to 0.095	0.002	1.231
Competence in digital technologies	−0.017	−0.066 to 0.033	0.504	−0.030	−0.084 to 0.024	0.268	1.235

^a^ R^2^ for the multivariable model = 3.7%; *p*-value for ANOVA = 0.010. CI: confidence interval; VIF: variance inflation factor.

**Table 5:** Linear regression models with the score on the personal advantages factor of the Artificial Intelligence in Mental Health Scale as the dependent variable (n = 276).

Independent Variables	Univariate Models			Multivariable Model ^a^			VIF
	Unadjusted Coefficient Beta	95% CI for Beta	*p*-Value	Adjusted Coefficient Beta	95% CI for Beta	*p*-Value	
Males vs. females	0.478	0.215 to 0.741	<0.001	0.548	0.277 to 0.820	<0.001	1.068
Age	−0.004	−0.015 to 0.007	0.506	−0.007	−0.019 to 0.005	0.238	1.185
Financial status	−0.023	−0.090 to 0.044	0.500	−0.033	−0.103 to 0.037	0.354	1.126
Daily use of social media/websites (hours)	0.017	−0.027 to 0.060	0.452	0.018	−0.030 to 0.065	0.462	1.231
Competence in digital technologies	−0.006	−0.069 to 0.057	0.853	−0.033	−0.101 to 0.036	0.347	1.235

^a^ R^2^ for the multivariable model = 4.2%; *p*-value for ANOVA = 0.005. CI: confidence interval; VIF: variance inflation factor.

**Table 6:** Linear regression models with the score on the acceptance factor of the Attitudes Towards Artificial Intelligence Scale as the dependent variable (n = 276).

Independent Variables	Univariate Models			Multivariable Model ^a^			VIF
	Unadjusted Coefficient Beta	95% CI for Beta	*p*-Value	Adjusted Coefficient Beta	95% CI for Beta	*p*-Value	
Males vs. females	1.607	1.031 to 2.184	<0.001	1.587	0.993 to 2.181	<0.001	1.068
Age	0.003	−0.022 to 0.027	0.818	−0.001	−0.026 to 0.025	0.959	1.185
Financial status	0.012	−0.140 to 0.163	0.879	−0.098	−0.250 to 0.055	0.209	1.126
Daily use of social media/websites (hours)	−0.020	−0.118 to 0.078	0.688	−0.046	−0.149 to 0.057	0.380	1.231
Competence in digital technologies	0.167	0.027 to 0.308	0.020	0.164	0.014 to 0.313	0.032	1.235

^a^ R^2^ for the multivariable model = 10.1%; *p*-value for ANOVA = <0.001. CI: confidence interval; VIF: variance inflation factor.

**Table 7:** Linear regression models with the score on the fear factor of the Attitudes Towards Artificial Intelligence Scale as the dependent variable (n = 276).

Independent Variables	Univariate Models			Multivariable Model ^a^			VIF
	Unadjusted Coefficient Beta	95% CI for Beta	*p*-Value	Adjusted Coefficient Beta	95% CI for Beta	*p*-Value	
Males vs. females	0.532	−0.016 to 1.079	0.057	0.772	0.231 to 1.312	0.005	1.068
Age	0.009	−0.014 to 0.031	0.445	0.011	−0.012 to 0.034	0.367	1.185
Financial status	−0.321	−0.453 to −0.189	<0.001	−0.329	−0.467 to −0.190	<0.001	1.126
Daily use of social media/websites (hours)	0.086	−0.003 to 0.175	0.057	0.071	−0.023 to 0.165	0.138	1.231
Competence in digital technologies	−0.057	−0.186 to 0.072	0.386	−0.040	−0.176 to 0.096	0.566	1.235

^a^ R^2^ for the multivariable model = 9.7%; *p*-value for ANOVA = <0.001. CI: confidence interval; VIF: variance inflation factor.

## Data Availability

Data are available at Figshare at https://doi.org/10.6084/m9.figshare.30600857.

## Supplementary Materials

The following supporting information can be downloaded at: https://www.mdpi.com/article/10.3390/nursrep16040133/s1, Figure S1: histogram of the residuals with the score on the “technical advantages” factor of the Artificial Intelligence in Mental Health Scale as the dependent variable; Figure S2: scatterplot of residuals versus predicted values with the score on the “technical advantages” factor of the Artificial Intelligence in Mental Health Scale as the dependent variable; Figure S3: histogram of the residuals with the score on the “personal advantages” factor of the Artificial Intelligence in Mental Health Scale as the dependent variable; Figure S4: scatterplot of residuals versus predicted values with the score on the “personal advantages” factor of the Artificial Intelligence in Mental Health Scale as the dependent variable; Figure S5: histogram of the residuals with the score on the acceptance factor of the Attitudes Towards Artificial Intelligence Scale as the dependent variable; Figure S6: scatterplot of residuals versus predicted values with the score on the acceptance factor of the Attitudes Towards Artificial Intelligence Scale as the dependent variable; Figure S7: histogram of the residuals with the score on the fear factor of the Attitudes Towards Artificial Intelligence Scale as the dependent variable; Figure S8: scatterplot of residuals versus predicted values with the score on the fear factor of the Attitudes Towards Artificial Intelligence Scale as the dependent variable.

## Public Involvement Statement

There was no public involvement in any aspect of this research.

## Guidelines and Standards Statement

This manuscript was drafted against the Strengthening the Reporting of Observational Studies in Epidemiology (STROBE) guidelines https://www.strobe-statement.org/ for observational research studies.

## Abbreviations

The following abbreviations are used in this manuscript:

AI	Artificial Intelligence
AIMHS	AI Mental Health Scale
ATAI	Attitudes Towards Artificial Intelligence Scale
LLMs	Large Language Models
PLMs	Pretrained Models
RAG	Retrieval Augmented Generation
SD	Standard Deviation
VIFs	Variance Inflation Factors
CIs	Confidence Intervals
