# Supplementary figures and images for: Exploring Nurses’ Perspectives on the Use of Artificial Intelligence Chatbots for Mental Health Support: A Cross-Sectional Study in Greece
Source: Nurs Rep. 2026 Apr 13;16(4):133. doi: 10.3390/nursrep16040133 (PMC13118656; doi:10.3390/nursrep16040133)

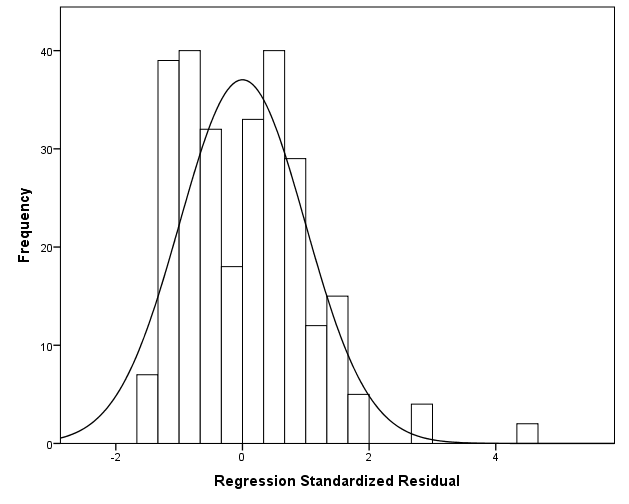

Supplement: Supplementary file 1 [file nursrep-16-00133-s001.zip › Supplementary Figure 1.png]

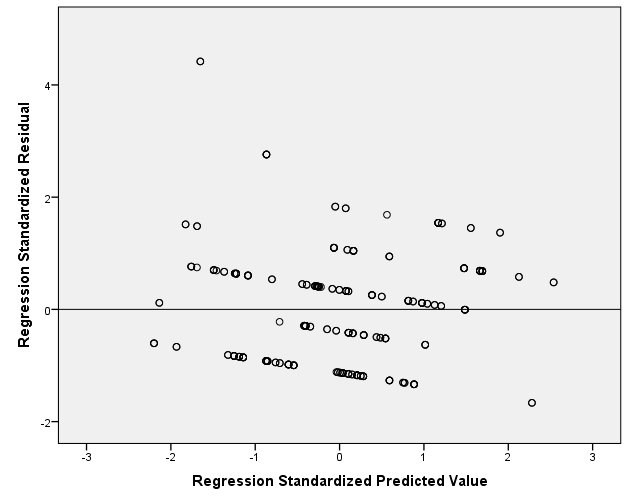

Supplement: Supplementary file 1 [file nursrep-16-00133-s001.zip › Supplementary Figure 2.png]

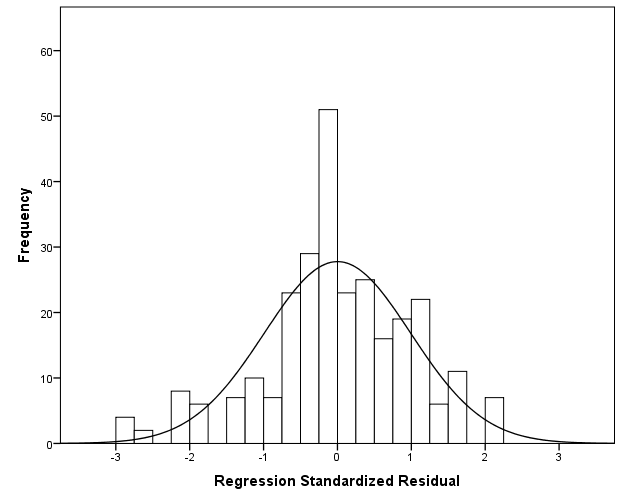

Supplement: Supplementary file 1 [file nursrep-16-00133-s001.zip › Supplementary Figure 3.png]

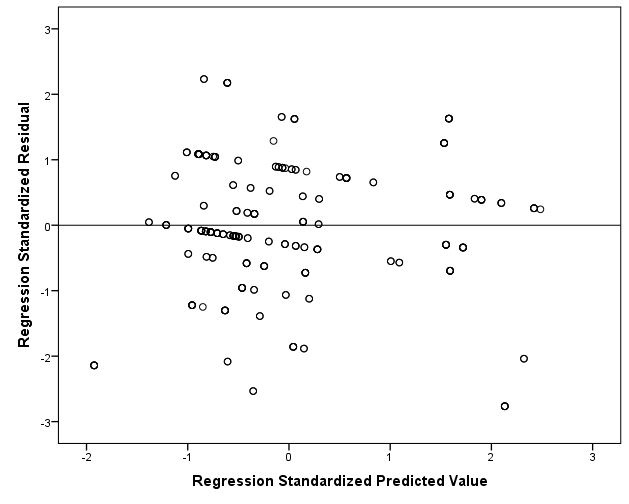

Supplement: Supplementary file 1 [file nursrep-16-00133-s001.zip › Supplementary Figure 4.png]

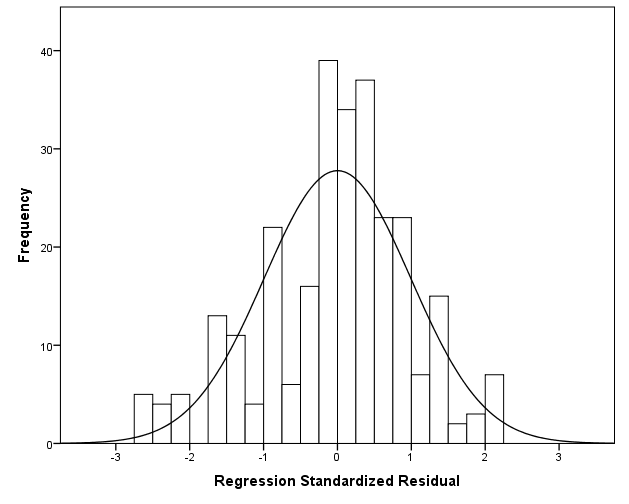

Supplement: Supplementary file 1 [file nursrep-16-00133-s001.zip › Supplementary Figure 5.png]

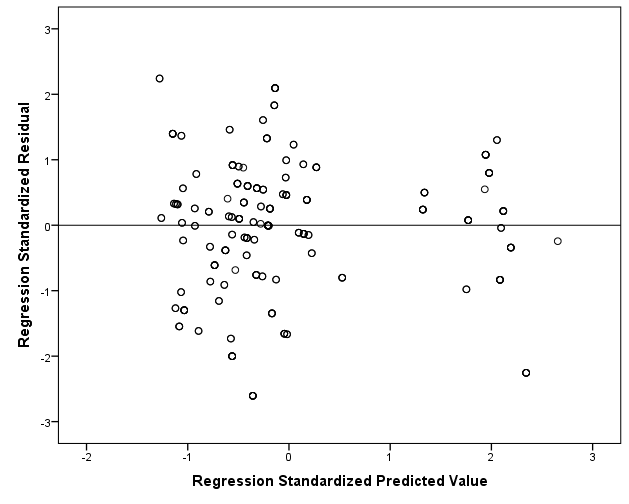

Supplement: Supplementary file 1 [file nursrep-16-00133-s001.zip › Supplementary Figure 6.png]

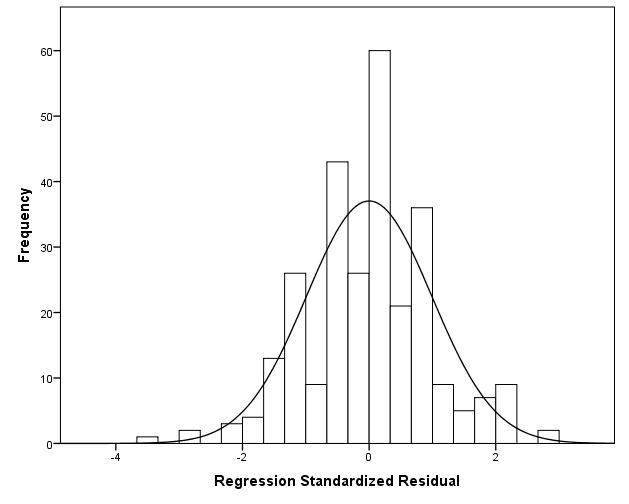

Supplement: Supplementary file 1 [file nursrep-16-00133-s001.zip › Supplementary Figure 7.png]

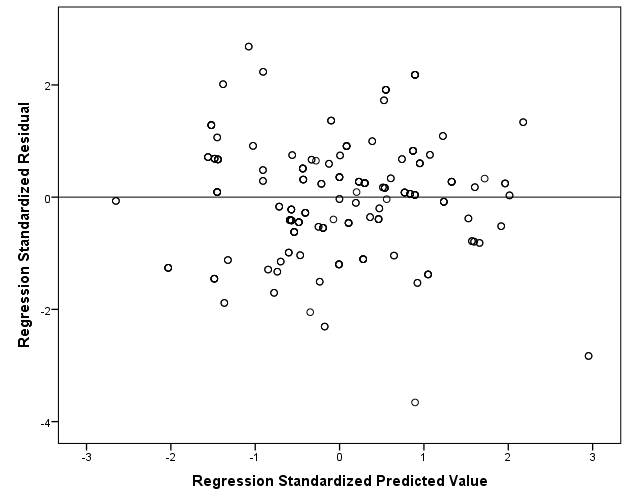

Supplement: Supplementary file 1 [file nursrep-16-00133-s001.zip › Supplementary Figure 8.png]
